# CAPAbility: comparison of the JOURNEY II Bi-Cruciate Stabilised and GENESIS II total knee arthroplasty in performance and functional ability: protocol of a randomised controlled trial

**DOI:** 10.1186/s13063-020-4143-4

**Published:** 2020-02-24

**Authors:** Celia Clarke, Valerie Pomeroy, Allan Clark, Graham Creelman, Nicola Hancock, Simon Horton, Anne Killett, Charles Mann, Estelle Payerne, Andoni Toms, Gareth Roberts, Toby Smith, Ann Marie Swart, Iain McNamara

**Affiliations:** 1 School of Health Sciences, University of East Anglia, Norwich, UK; 2 Norwich Medical School, University of East Anglia, Norwich, UK; 3 Patient and Public Involvement, Norwich, UK; 4 Department of Trauma and Orthopaedics, Norfolk and Norwich University Hospital, Norwich, UK; 5 Norwich Clinical Trials Unit, UEA, Norwich, UK; 6 Norwich Radiology Academy, Norfolk and Norwich University Hospital, Norwich, UK; 7 Nuffield Department of Orthopaedics, Rheumatology and Musculoskeletal Sciences, University of Oxford, Oxford, UK

**Keywords:** Total knee arthroplasty, Knee replacement, Functional ability, Knee prosthesis, Kinematics, Primary osteoarthritis

## Abstract

**Background:**

Osteoarthritis of the knee is a common condition that is expected to rise in the next two decades leading to an associated increase in total knee replacement (TKR) surgery. Although there is little debate regarding the safety and efficacy of modern TKR, up to 20% of patients report poor functional outcomes following surgery. This study will investigate the functional outcome of two TKRs; the JOURNEY II Bi-Cruciate Stabilised knee arthroplasty, a newer knee prosthesis designed to provide guided motion and improve knee kinematics by more closely approximating a normal knee, and the GENESIS II, a proven existing design.

**Aim:**

To compare the change in Patient-reported Outcome Measures (PROMs) scores of the JOURNEY II BCS and the GENESIS II from pre-operation to 6 months post operation.

**Methods:**

CAPAbility is a pragmatic, blinded, two-arm parallel, randomised controlled trial recruiting patients with primary osteoarthritis due to have unilateral TKR surgery across two UK hospitals. Eligible participants (*n* = 80) will be randomly allocated to receive either the JOURNEY II or the GENESIS II BCS knee prosthesis. Baseline measures will be taken prior to surgery. Patients will be followed at 1 week, 6 to 8 weeks and 6 months post-operatively. The primary outcome is the Oxford Knee Score (OKS) at 6 months post-operatively. Secondary outcomes include: other PROMs, biomechanical, radiological (computerised tomography, (CT)), clinical efficacy and safety outcomes. An embedded qualitative study will also investigate patients’ perspectives via interview pre and post surgery on variables known to affect the outcome of TKR surgery. A sub-sample (*n* = 30) will have additional in-depth interviews to explore the themes identified. The surgeons’ perspectives on the operation will be investigated by a group interview after all participants have undergone surgery.

**Discussion:**

This trial will evaluate two generations of TKR using PROMS, kinematic and radiological analyses and qualitative outcomes from the patient perspective.

**Trial registration:**

International Standard Randomised Controlled Trials Number Registration, ID: ISRCTN32315753. Registered on 12 December 2017.

## Introduction

### Background and rationale

Osteoarthritis of the knee is a common musculoskeletal condition. The surgical management of painful, end-stage osteoarthritis is by total knee replacement (TKR) which should be considered before there is prolonged and established functional limitation and severe pain [1]. Over 100,000 TKRs were performed in the UK in 2019 [2]. While TKR frequently reduces pain and improves physical function in the majority of patients, 20% of patients report poor functional outcomes post-operatively [3, 4]. Such poor outcomes are of importance to patients and have a considerable financial and service-provision impact on NHS care. Research is needed to improve post-arthroplasty outcomes for those patients.

There is a paucity of literature regarding the kinematic outcomes of patients following TKR. However, there is uncertainty as to whether good Patient-reported Outcome Measures (PROMs) are associated with a return to normal kinematics of the TKR knee compared to the native knee. Movement analysis can be used to examine the change in kinematics before and after TKR by examining functional movements in activities of daily living.

The long-term success of TKR depends largely on correct component alignment and accurate ligamentous balancing [5]. The impact of femoral- and tibial-component rotation on flexion-gap balance, patellofemoral tracking and normal kinematic function is well-known [6–8]. Complications secondary to poor component alignment have been reported to lead to a higher rate of revision surgery [9, 10]. Computerised tomography (CT) imaging is a valid and reproducible technique for accurately measuring TKR-component rotation [11, 12]. However, despite CT being widely used to examine implant rotation, the correlation between rotational alignment, PROMs and kinematic function comparing pre- and post-operative measurement is unclear [13, 14]. It is hypothesised that patients with poor rotational profile post-operatively compared to their pre-operative values will have significantly worse PROMs, movement parameters and patient satisfaction.

We report the protocol of a two-group, parallel randomised controlled trial (RCT) comparing patient-reported, surgical and biomechanical outcomes from a TKR of a newer design (the JOURNEY II Bi-Cruciate Stabilised knee arthroplasty (BCS)) designed to provide improved kinematic outcomes compared to an older design TKR implant (the GENESIS II).

This protocol (version 2.4, dated 27 February 2019) has been written and reported according to the Standard Protocol Items: Recommendations for Interventional Trials (SPIRIT) guidance and Checklist [15] (see Additional file 1: SPIRIT 2013 Checklist).

### Aims

The principal aim of the trial is to compare the change in PROMs scores of the JOURNEY II BCS knee and the GENESIS II knee from pre-operation to 6 months post operation. Additional aims are as follows:

1. To determine whether the temporal and spatial parameters of gait, the range of movement and static and dynamic balance are closer to aged-matched normative data in those receiving the JOURNEY II BCS compared to those receiving the GENESIS II knee
2. To monitor the change in function (Aim 1 above) and PROMs of the JOURNEY II BCS and the GENESIS II knee from post operation to 6 months post operation
3. From CT scan measures, determine anatomical landmarks and rotational profile around the native knee and following TKR to ascertain the component rotational position post-operatively compared to anatomical landmarks
4. To examine the relationship between rotational values determined by CT scanning with pre- and post-operative PROMs and movement analysis
5. To develop knowledge and understanding of patient and surgeon experiences, perspectives and satisfaction when receiving or implanting the JOURNEY II BCS compared with the GENESIS II knee, and their experiences of recovery and rehabilitation

## Methods: participants, interventions and outcomes

### Trial design

This is a pragmatic, triple-blinded, parallel, superiority, randomised controlled trial of the JOURNEY II BCS (intervention) versus GENESIS II (control) in patients with primary osteoarthritis undergoing TKR. Embedded in the clinical trial is a qualitative investigation of participants’ confidence in the TKR received and their experiences of the recovery process in the first 6 months after surgery. The aim of this is to identify any differences in the experience of recovery between each type of TKR. Surgeons will also be interviewed to investigate their perceptions of the surgery and patient’s rehabilitation.

**Fig. 1:**
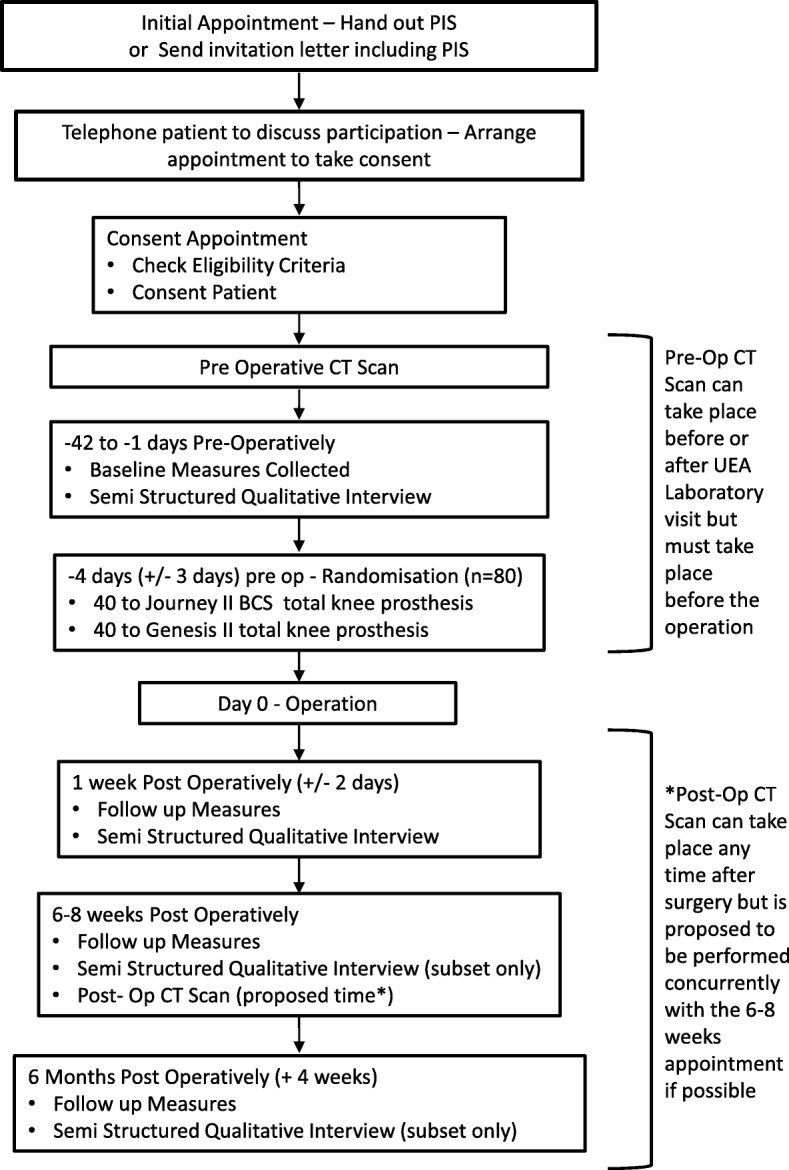
Comparison of the JOURNEY II Bi-Cruciate Stabilised and GENESIS II total knee arthroplasty in performance and functional ability (CAPAbility) trial outline

The trial outline is illustrated in Fig. 1.

### Study setting

Trial sites were pre-selected on the basis of their locality to facilitate data collection (namely the kinematic assessment). Sites include the Norfolk and Norwich University Hospital (NNUH), where all patients recruited to the trial will be referred for consideration of TKR. The NNUH refers a proportion of its TKR patients to Spire Norwich where the operation and follow-up physiotherapy is delivered. Both hospital are participating in this trial. All CT scans will be performed at NNUH. The biomechanical assessment will be undertaken in a specialist movement analysis laboratory (MoveExLab) at the University of East Anglia (UEA).

### Eligibility criteria

To be eligible for the trial, patients must satisfy the surgeon’s general requirements for a TKR, meet all inclusion criteria and none of the exclusion criteria listed in Table 1.

**Table 1:** Eligibility criteria

Inclusion criteria	
• Listed for a primary TKR at the NNUH (may be referred to Spire Norwich for the operation)	
• Indication for the TKR is primary osteoarthritis of the knee joint involving one or more compartments	
• Aged 18 years or over	
• Patient willing to provide full informed consent to the trial including consent for any incidental findings to be communicated to their general practitioner (GP)	
Exclusion criteria	
• Listed for a single-stage, bilateral TKR procedure	
• Severe symptoms in the contralateral knee so as to require staged, bilateral knee replacements within 6 months of the primary procedure	
• Fixed-flexion deformity of 15° or greater or patients who may require excessive resection of the distal femur	
• Clinically assessed uncorrectable varus/valgus deformity of 15° or greater	
• Any co-morbidity which, in the opinion of the investigator, is severe enough to present an unacceptable risk to the patient’s safety	
• Inflammatory arthritis	
• Previous septic arthritis in the affected knee joint	
• Previous surgery to the collateral ligaments of the affected knee	
• A contralateral TKR that has been implanted less than 1 year from the date of consultation, or severely painful	
• Patients taking warfarin or Novel Oral Anti-Coagulants	
• Will not be resident in the catchment area for NNUH for at least 6 months post surgery	
• Undertaking the surgery as a private (non-NHS) patient	
• Patients who, in the opinion of the clinical staff, do not have capacity to consent	
• Patients who are pregnant	
• Unable to understand written and spoken English	
• Patients currently enrolled on an interventional trial involving surgery, exercise or rehabilitation. Patients can be co-enrolled into studies not meeting the above criteria given prior agreement from the TMG of both studies. Patients who enter the study are eligible for entry onto the National Joint Registry and in terms of the Journey II BCS, into Beyond Compliance	

Patients will be excluded if they are currently enrolled on an interventional trial involving surgery, exercise or rehabilitation. Patients can be co-enrolled into studies given prior agreement from the Trial Management Group (TMG) of both studies. Patients who enter the study are eligible for entry onto the UK National Joint Registry.

### Screening

Potential participants will be approached via a single route. Potential participants will be screened by a member of the clinical team in collaboration with research nurses after having been added to the orthopaedic clinic waiting list. Potentially eligible patients who meet the eligibility criteria will either be handed a Patient Information Sheet (PIS) if still at the clinic, or be posted an invitation letter informing them that the trial is taking place and include the PIS. After having been provided the trial PIS, potential participants will be telephoned by a research nurse. To minimise the possibility of attrition, appointments for outcome measures will be agreed with participants when they enter the trial. In addition, members of the research team will maintain regular contact with participants to ensure attendance at follow-up visits and to monitor any adverse events (AEs).

### Informed consent

Written informed consent to enter and be randomised into the trial will be taken by a member of the clinical team and obtained from participants after explanation of the aims, methods, benefits and potential hazards of the trial. Potential participants will be given as much time as they need to consider whether or not to provide informed consent. Consent will take place before any trial-related measures, at a time convenient to the potential participant, preferably at a time to combine with one or more of the measures to reduce participant visits.

If a participant withdraws prior to surgery, an additional participant will be randomised to ensure that 80 participants complete the surgery.

Patients who, in the opinion of the clinical team, do not have capacity to consent, will be ineligible. If a participant loses capacity during the course of the trial, they will be withdrawn from any further assessments, but any data already collected will be retained. Consent will be re-sought if new information becomes available that affects the participant’s consent in any way. This will be documented in a revision to the PIS and the participant will be asked to sign an updated consent form. These will be approved by the Ethics Committee prior to their use. A copy of the approved consent form is available from the Norwich Clinical Trials Unit (NCTU).

### Sample size

Eighty patients will be recruited onto this superiority trial. The sample size has been calculated from the Oxford Knee Score (OKS) [16]. The OKS ranges from a score of 12 to 60, with 12 being the best outcome. The minimally important clinical difference for OKS is 5 [17, 18] and a standard deviation of 7.4 [19]. For an 80% power, and an assumed dropout rate of 10%, 80 participants will be randomised to one of the two groups.

### Participant timeline

The participant timeline is shown in Fig. 1. Where possible, trial visits will be combined with standard clinic visits. Should additional visits be necessary, participants will be reimbursed for travel costs.

### Interventions

All participants will receive routine care provided by the NHS. Pre-operative and peri-operative care is standardised irrespective of implant.

### Explanation for choice of comparators (Genesis II versus JOURNEY II BCS)

The GENESIS II TKR system made by Smith and Nephew (Smith and Nephew plc, Watford, UK) is frequently used in standard practice within the NHS [2]. It has been the standard TKR within the NNUH and Spire Norwich Hospitals for over 10 years. The Genesis II has a survivorship of over 93% of implants at 15 years [2, 20] and offers good health-related quality of life outcomes [21].

A newer device, JOURNEY II BCS, also manufactured by Smith and Nephew, has been developed to theoretically provide improved kinematic outcomes compared to the GENESIS II [22]. These improvements are proposed to include:

- Alteration in the dimensions of the femoral component to reduce soft-tissue strain and maintain more natural translation and external rotation
- Reduction in the thickness of the lateral and medial anterior flange of the femoral component and edge tapering to reducing tension on the iliotibial band (ITB) and iliotibial-patellar bands (ITPB)
- Reduction in the width of the femoral component to limit implant overhang, and reduction in the mid-flexion thickness of the medial condyle to maintain more consistent strain on the medial collateral ligament (MCL) throughout the flexion range
- A superior cam position, which serves to decrease femoral rollback in the targeted ranges of motion, increase femoral external rotation, and lower the point of tibial post contact in deep flexion

While there is fluoroscopic data to support normal kinematics in early and late flexion [23], there is a paucity of evidence exploring these hypotheses for this newer implant.

### Surgical flow and training

Surgeons will be high-volume arthroplasty surgeons who work at both NNUH and Spire Norwich Hospital. The standard implant at both sites is the Genesis II TKR system. All surgeons have used this implant for many years and are very familiar with the surgical technique. All surgeons and theatre staff have received training on the implantation of the JOURNEY II BCS implant. All surgeons have undergone training on the JOURNEY BCS II implant in a cadaveric laboratory and have also undertaken a learning curve with the device until they were confident with the technique. This was supported by a Smith and Nephew representative. There are minimal differences in the surgical cuts and technique between the Genesis II and the JOURNEY BCS II. Participating surgeons felt that there was a shallow learning curve to the JOURNEY BCS II. Both devices are CE-marked and will be used within indication. Smith and Nephew are providing the JOURNEY II BCS at the same price as the GENESIS II system for this study.

### Surgical procedures

Devices will be identified and prepared for the operation by a surgical technician at the surgery site.

Participants allocated to the intervention device will receive the JOURNEY II BCS prosthesis while participants allocated to the control condition will receive the GENESIS II prosthesis. The type of device implanted and its serial number will be recorded on the trial database, by an unmasked member of the research team.

The surgical procedure will follow the standardised surgical approach and technique. It will be undertaken through a medial parapatellar approach. In both implants and in every case to ensure standardisation of technique, a posterior stabilised prosthesis with patella resurfacing will be used.

It is possible that a decision will be taken prior to, or during, the operation not to use the allocated device if, in the opinion of the surgeon, the patient is found to have become unsuitable for continued participation in the trial. The reasons for an allocated device not being used will be recorded on the trial database. In this case or if a participant chooses to withdraw consent for treatment, or follow-up, all data collected up to the point of withdrawal will be retained. The standard Norwich Enhanced Recovery Programme (NERP) [24, 25] is used for the anaesthetic technique and post-operative recovery.

### Post-operative rehabilitation

Post-operative rehabilitation will follow routine clinical care at NNUH and Spire Norwich [24, 25]. While an inpatient, participants will be seen by a physiotherapist for routine care at least twice daily to progress on a tailored gait re-education and exercise programme during their hospital admission. This will be recorded in an in-patient hospital rehabilitation log. Once safe for discharge, patients will be asked to continue a home exercise programme and gait re-education. This will consist of daily (advised) knee-flexion range-of-motion exercises and quadriceps strengthening.

At week 4 post-operatively, all participants will attend an exercise-group-based intervention delivered by a qualified physiotherapist and a physiotherapy assistant. These sessions will be used to increase participant’s knee range of motion, strength and overall confidence to undertake more strenuous exercises. Participants will attend this class weekly for two to six sessions depending on their need. All rehabilitation interventions will be recorded in a post-discharge rehabilitation log. Participants will be encouraged to continue their exercises which are prescribed within the group as part of a home-exercise programme.

No additional ancillary or post-trial care will be provided (in the absence of AEs) to trial participants.

### Outcomes

The schedule of enrolment, interventions and assessment is shown in Table 2. The PROMs will be administered by research nurses apart from the week-1 follow-up telephone call undertaken by the research associate performing the qualitative interview. The CT scans will be performed at the NNUH by research radiographers and reported by a consultant radiologist. The biomechanical assessments and qualitative interviews will be performed at the MoveExLab at the UEA. Participants who were unable to attend an assessment appointments were provided with an alternative appointment. If participants were unable to attend any alternative assessment appointments, PROMs data was collected during a telephone call to promotion participant retention and follow-up.

**Table 2:**
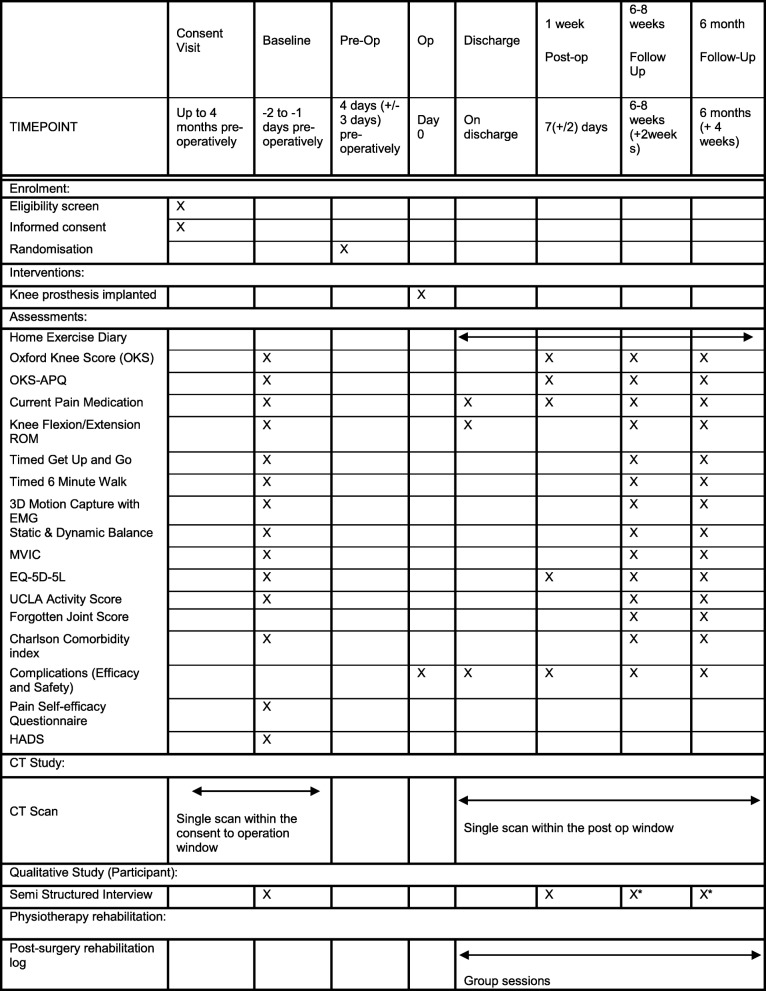
Schedule of enrolment, interventions and assessments

*subset of 30 patient; *CT* computerised tomography, *HADS* Hospital Anxiety and Depression Score, *MVIC maximum voluntary* isometric contraction, *OKS* Oxford Knee Score, *OKS-APQ* Oxford Knee Score Activity and Participation Questionnaire, *Pre-Op* pre-operative, *Post-Op* post-operative, *ROM* range of motion, *UCLA* University of California Los Angeles

### Primary outcome

The OKS [16] will be used to assess patient-reported functional status at 6 months post surgery.

#### Secondary outcomes: Patient-Reported Outcome Measures

The Oxford Knee Score (OKS) [16] – Activity and Participation Questionnaire (OKS-APQ), [26] EuroQol 5 dimensions, 5 levels health survey (EQ-5D-5 L) [27], UCLA Activity Score [28], Hospital Anxiety and Depression Score (HADS) [29], Forgotten Joint Score (FJS) [30], and 2-Item Pain Self-Efficacy Questionnaire [31].

#### Secondary outcomes: clinical efficacy outcomes

Clinical efficacy will be evaluated by:

- Surgical-related parameters: need for revision surgery; length of hospital stay and change in pain medication will be collected during in-patient stay and at all the follow-up time points
- Performance-related parameters: knee flexion and extension ranges of movement, measured at 6 to 8 weeks and 6 months post-operatively by the research associate in the MoveExLab (and by the research physiotherapist at baseline as part of routine care); timed-up-and-go (TUG) [32] and timed 6-minute walk test [33] recorded at the 6–8 week and 6-month time points by the research associate in the MoveExLab

#### Secondary outcomes: clinical safety outcomes

Complications related to the surgery (e.g. anaesthesia-related problems, bleeding, morbidities) will be collected from a notes review, prior to discharge, post-discharge, rehabilitation and follow-up. Additionally, at each visit, participants will be asked whether they have received additional treatment since their surgery/previous visit and what that consisted of.

#### Secondary outcomes: biomechanical outcomes

All biomechanical measures will be collected in the MoveExLab by the research associate. Three-dimensional motion capture using eight cameras (Vicon Motion System, Oxford, UK), three built in force plates (Bertec Corporation, Columbus, OH, USA) and surface electromyography (EMG) (Delsys, Natick, MA, USA). Participants will be unshod and asked to walk at their self-selected speed. A minimum of three heel strikes from each foot will be used to construct an average.

1. Overground walking: unshod and walking at self-selected speed:
  - Spatiotemporal parameters; speed, cadence, step-length, stride-length and symmetry
  - Kinematics of bilateral hip, knee and ankle joints
  - Kinetics: moments of bilateral hip, knee and ankle joints and ground reaction forces during the stance phase
  - EMG parameters: recruitment patterns of quadriceps: rectus femoris, vastus medialis and vastus lateralis, hamstrings: semitendinosus, biceps femoris, tibialis anterior, medial and lateral gastrocnemius
2. Stair ascent and descent:
  - Spatiotemporal parameters; speed, cadence, symmetry
  - Kinematics of bilateral hip, knee and ankle joints
  - Kinetics: moments of bilateral hip, knee and ankle joints and ground reaction forces from the bottom step

Static balance measures will be completed on a single, in-built force plate (Bertec Corporation, Columbus, OH, USA). Participants will be instructed to stand with their feet shoulder-width apart for double stance with their eyes closed and then open for 10 s. Three attempts will be recorded. Participants will then be instructed to stand on one leg in the centre of the force plate with their hands on their hips with their eyes open and closed for 10 s. Each limb will be tested. Three trials of 10 s will be recorded. The time will be stopped if the participant places the other foot on the floor. Each participant will be given six attempts at each position.

3. Static balance; measures of centre of pressure (CoP) from single- and double-leg standing
  - Anterior-posterior (AP), medial-lateral (ML) and CoP path length
  - AP, ML and CoP velocity
  - AP, ML and CoP range and standard deviation (SD)
4. Time-to-boundary (TTB) [34]
  - TTB minimum, mean and SD
5. Modified Star Excursion Balance Test (mSEBT) [35]
  - Anterior, posteromedial and posterolateral distance (millimetres) on both limbs

#### Secondary outcomes: radiological outcomes

##### Radiographs

Pre-operative and post-operative conventional semi-flexed AP and lateral radiographs of the knee will be acquired.

##### Computerised tomography

A rotational-profile CT protocol will be acquired at the NNUH Radiology Department under standard operating procedure.

This will consist of three separate axial acquisitions through the femoral necks, knees and ankles reconstructed on bone and soft-tissue algorithms. The images through the knee will be split into two acquisitions according to the Berger protocol [36]. The pre-operative CT will be performed in the time after consent for the study and before TKR. The post-operative CT is not time sensitive and will be performed any time following surgery.

Two independent observers, radiologists under direct supervision of a senior musculoskeletal radiologist, will obtain the following measurements from the CT. In the case of disagreement between the two independent observers, through discussion, the senior musculoskeletal radiologist will act as adjudicator to ensure that agreement is met. Measurements will include:

##### Pre-operative

1. Femoral ante-torsion (degrees)
2. Tibial tubercle-trochlear groove distance (TT-TG) (millimetres)
3. Tibial torsion (degrees)

##### Post-operative

1. Femoral ante-torsion (degrees)
2. Femoral-component version (degrees)
3. Tibial-component version (degrees)
4. Tibial torsion (degrees)

In the event of an incidental finding being reported, the clinical chief investigator will organise the necessary clinical follow-up which may include referral to an appropriate clinician and the organisation of further investigations.

#### Secondary outcomes: qualitative study

Interviews will be completed either via a telephone call or face-to-face by the research associate. This flexibility was adopted to promote participant retention and complete follow-up. These interviews will be audio-recorded and transcribed for analysis.

All TKR participants will be invited to take part in an interview and complete a self-efficacy questionnaire and the HADS at baseline and a telephone call interview at the 7 days (± 2 days) surgery.

Two additional post-surgery interviews will be carried out with a purposive sample of participants (*n* = 30), drawn equally from the intervention and control groups. Sampling decisions will be based on the following factors: age; sex; ethnicity; socioeconomic status; OKS; self-efficacy; expectations, mood and symptom management (as ascertained from inspection of baseline interviews).

The aims of the interviews are to gain in-depth understanding of patient perspectives on important variables known to affect outcomes of TKR surgery [4, 37–39]. Specific themes will be:

1. To explore patients’ expectations of and hopes for surgery (pre-operative only)
2. To explore patients’ experiences and perspectives on: mood, pain and function – everyday mobility, participation in work, social roles and activities; surgery and post-operative clinical management; rehabilitation and recovery, and social support

All surgeons will be invited to consent to a face-to-face interview after the last participant’s surgery to explore their perspective on using each prostheses and their overall experience of surgery.

## Methods: assignment of interventions

### Allocation

An interactive web-randomisation system will be used by a member of the research team who is not blinded to the intervention. Participants will be randomly assigned to either control or experimental group with a 1:1 allocation as per a computer-generated randomisation schedule. Randomisation will occur after the completion of all baseline tests. This will take place 4 days (± 3 days) prior to the operation to allow the correct TKR to be made available. Randomisation will be stratified by: (1) site (i.e. hospital where surgery is to take place); and (2) age (< 60 years = younger; equal or ≥ 60 years = older) [40, 41].

### Blinding (masking)

It is not possible to blind the surgeon to the trial intervention. However, the participants, the physiotherapists and all staff involved in assessing outcomes will be blinded. Processes will be in place to maintain blinding. These will include concealment in a sealed envelope of the surgery notes mentioning the prosthesis implanted in the patient file.

In the unlikely event of a research nurse accidentally becoming unmasked, the contacts, assessments and data entry for that participant will be undertaken by another member of the research team for the remaining period of trial participation for that participant. Accidental unmasking will be logged and monitored to ensure that the appropriate steps are taken to prevent a re-occurrence.

The clinical staff providing usual care will also be blinded. The decision to unmask a case will be made when knowledge of an individual’s allocated treatment is required to enable treatment of a serious adverse event (SAE) which is likely to be caused by the type of device implanted.

Where possible, requests for emergency unmasking of individuals will be made via the trial manager in agreement with the clinical chief investigator. However, in circumstances where there is insufficient time to make this request or for agreement to be sought, the treating clinician can make the decision to unmask immediately. This can be done via the trial database.

## Methods: data management and analysis

### Data management

Each participant will be given a unique trial Participant Identification Number (PIN). Data will be entered under the participant’s PIN number onto the central database stored on the servers based at NCTU. Access to the database will be via unique, individually assigned (i.e. not generic) usernames and passwords, and only accessible to members of the CAPAbility trial team at NCTU, and external regulators if requested. The servers are protected by firewalls and are patched and maintained according to best practice. The physical location of the servers is protected physically and environmentally in accordance with UEA’s General Information Security Policy 3 (GISP3: Physical and environmental security).

The database and associated code have been developed by NCTU Data Management, in conjunction with the CAPAbility trial team. The database software provides a number of features to help maintain data quality, including; maintaining an audit trail, allowing custom validations on all data, allowing users to raise data-query requests and search facilities to identify validation failure/missing data. After completion of the trial, the database will be retained on the servers of NCTU for on-going analysis of secondary outcomes.

The identification, screening and enrolment logs, linking participant identifiable data to the pseudoanonymised PIN, will be held locally by the trial site. This will either be held in written form in a locked filing cabinet or electronically in password-protected form on hospital computers. After completion of the trial, the identification, screening and enrolment logs will be stored securely by the sites for 15 years unless otherwise advised by NCTU. The consent form will explain that if a participant wishes to withdraw from the study the data acquired prior to that point will be retained. Reason for withdrawal will be recorded, if given, as will loss to follow-up.

### Statistical analysis

A full Statistical Analysis Plan (SAP) will be developed between the trial statistician and chief investigators and agreed with the trial’s Governance Committees. All analysis will be based on the intention-to-treat principle in which all participants will be analysed according to the group to which they were allocated, regardless of compliance.

Baseline factors will be summarised by group. All continuous variables will be summarised by the mean and SD, or if appropriate, the median and interquartile range. Categorical variables will be summarised with the number and percentage, in each category.

The primary comparison for OKS will be made using a general linear model with the stratification factors included as fixed effects. The difference between arms will be summarised using the mean difference, with 95% confidence intervals presented. A similar analysis will be undertaken for all other outcome measures.

For the temporal gait parameters and kinematic outcomes, each participant’s ‘closeness’ to age-matched normative data will be calculated. This will then be compared between groups using a general linear model with the stratification factors included as fixed effects. This data will also be presented graphically via scatter and distributional graphs to describe the deviations from the normative data.

For all the measures of movement listed, a general linear model with the stratification factors included as fixed-effects will be used to assess for between-group differences. If appropriate, adjusted analyses will be undertaken by including baseline factors and fixed effects in the above models.

### Assumptions and sensitivity analysis

All the assumptions will be checked via distribution graphs and tests. If the assumptions are not valid, transformation will be considered. If none are found, a non-parametric approach will be used. The pattern of missing or incomplete data will be assessed. If appropriate, missing data will be imputed. The baseline comparability of the groups will be assessed. If appropriate, any factor found to be imbalanced and important, will be adjusted for in the analysis.

Exploratory subgroup analysis will be undertaken by including an interaction in the model to assess whether the effectiveness of the prosthesis is dependent on age or gender.

All analyses will be conducted using Stata and the full SAP will be produced, and approved, before any comparative analysis is undertaken.

### Additional analyses – CT scans

All rotational profile measurements will be performed at NNUH under standard operating procedure on a full diagnostic workstation (Synapse DICOM viewer, Fujifilm, Japan; High resolution 2 K monitors, Radiforce RX340, Eizo, Mönchengladbach, Germany) in the bioimaging laboratory and under the supervision of a consultant musculoskeletal radiologist (AT).

### Reproducibility

Inter-rater reliability will be assessed using intra-class correlation coefficients and 95% limits of agreement derived from Bland-Altman plots.

### TKR alignment versus native landmarks

The difference between the post-operative component rotational alignment and the pre-operative native landmarks will be assessed using Bland-Altman plots.

### Correlation with PROMS

The correlation between the PROMs and the difference between the post-operative component rotational alignment and the pre-operative native landmarks will be assessed using a correlation coefficient. A regression model will also be fitted including the randomisation group to allow for a potential between-group difference in PROMs.

### Correlation with movement analysis

A similar analysis will be undertaken for the correlation between movement analysis and the difference between the post-operative component alignment and the pre-operative native landmarks.

### Additional analyses – qualitative study

Interview transcripts will be organised using NVivo qualitative data management software (QSR International, Burlington, MA, USA). Analysis will follow qualitative content analysis procedures [42]. Coding and thematic analysis will be carried out independently by two experienced qualitative researchers. Trustworthiness strategies [43] will be used to increase the credibility, dependability and transferability of analysis and interpretation. This will include cross-checking and review of codes and themes; constant comparative method (hypothesis testing within and across the dataset) and deviant case analysis (the use of ‘outliers’ as a resource for understanding and interpretation of data) [44].

### Analysis population and missing data

The analysis population is defined as:

- Intention-to-treat: all randomised individuals
- Per-protocol: all randomised individuals who do not have an alternative TKR during the follow-up period. Individuals will be included up to the point of the alternative TKR
- Safety population: all randomised individuals who receive the TKR

Missing outcome data will be multiple imputed to increase precision of the treatment effect estimates. Sensitivity analyses will be conducted to assess the impact of the multiple imputations and a complete case analysis will also be conducted. All imputations will be examined to ensure that sensible values are being generated. Imputation models will contain baseline measures, outcome measures and factors predictive of missing data.

No interim analysis is planned for this study.

## Methods: monitoring

### Data monitoring

A TMG has been convened to assist with developing the design, co-ordination and strategic management of the trial. A Safety Committee will review safety data and act in place of a Data Monitoring Committee (DMC). Monitoring activities will be undertaken both centrally and on site. The frequency, type and intensity of routine and triggered monitoring are detailed in the Quality Management and Monitoring Plan (QMMP). Ongoing central monitoring will ensure quality and consistency of data thorough the trial. Details about data collection and cleaning are described in the Data Management Plan (DMP).

### Harms

#### Safety

Definitions of harm of the EU Directive 2001/20/EC Article 2 based on the principles of International Council for Harmonisation (ICH) guideline for Good Clinical Practice (GCP) apply to this trial. A record of all study-related SAEs, including details of the nature, onset, duration, severity, relationship to the device, relationship to the operative procedure, outcome and expectedness will be made on the relevant section(s) of the trial-specific SAE Form to be sent to the trial manager for onward reporting where required. SAEs resulting from surgery or arthroplasty complications (clinical and safety outcomes) will be reported in the relevant section of the Case Report Form (CRF).

All non-serious AEs and adverse drug events (ADEs), whether expected or not, should be recorded in the participant’s medical notes and also reported in the relevant section of the CRF.

Adverse events do *not include*:

- Readmissions for revision surgery
- Mild (i.e. not lasting for more than 5 days) anaesthetic-related complications: nausea, vomiting, dizziness, drowsiness, vaso-vagal drop, hypotension and constipation
- Medical or surgical procedures; the condition that led to the procedure is the AE
- Pre-existing disease or a condition present that was diagnosed before trial entry and does not worsen
- Hospitalisation where no untoward or unintended response has occurred, e.g. elective surgery, social admissions

The Safety Committee will be provided with safety data for each treatment arm including related AEs. The Committee will advise on the continuation or early stoppage of the trial in the unlikely event that there are concerns over harm to participants. The medical care in response to any harm from the trial participation will be managed by routine NHS care.

#### Auditing

The quality assurance (QA) and quality control (QC) considerations for the CAPAbility trial are based on the standard NCTU Quality Management Policy that includes a formal risk assessment, and that acknowledges the risks associated with trial conduct and proposals of how to mitigate them through appropriate QA and QC processes. Risks are defined in terms of their impact on: the rights and safety of participants; project concept including trial design, reliability of results and institutional risk; project management; and other considerations.

NCTU staff will review CRF data for errors and missing key data points. The trial database will also be programmed to generate reports on errors and error rates. Essential trial issues, events and outputs, including defined key data points, will be detailed in the trial DMP. The frequency, type and intensity of routine and triggered on-site monitoring will be detailed in the QMMP. The QMMP will also detail the procedures for review and sign-off of monitoring reports. In the event of a request for a trial-site inspection by any regulatory authority, NCTU must be notified as soon as possible.

### Ethics and dissemination

#### Research ethics approval

The trial is being conducted in accordance with CODEX rules and guidelines for research and the Helsinki Declaration as well as the ICH Guideline for GCP. The study protocol was approved by the East of England – Cambridge Central Research Ethics Committee (reference 16/EE/0230) prior to the start of the trial. The trial is registered on the International Standard Randomised Controlled Trials Number (ISRCTN) registry (reference ISRCTN32315753). Approval was granted by the Health Research Authority (HRA) and Confirmation of Capacity and Capability to conduct the trial has been provided by the NNUH Research and Development Office.

The NNUH is the trial sponsor and has delegated responsibility for the overall management of the trial to the co-chief investigators (Co-CIs) and NCTU including the trial design, co-ordination, monitoring and analysis and reporting of results. The standard procedures and policies at NCTU, a UK Clinical Research Collaboration (UKCRC)-registered trial unit and the study’s QMMP are followed. A TMG, including lay membership, has been formed to assist with the design, co-ordination and strategic management of the trial. An independent Safety Committee has also been set up to provide oversight on the trial and to safeguard the interests of the participants.

### Protocol amendments

The protocol was amended in August 2017 (before trial start at sites) to improve consistency and clarity. To that effect, an additional inclusion criterion was added to match the consent form requiring participants to agree to any incidental findings to be reported to their GP. The exclusion criteria relating to the use of the warfarin was also improved by the addition of novel anti-coagulants therapies which are increasingly being used. As part of this amendment we also changed the stratification criteria from American Society of Anesthesiologists (ASA) grade [45] and age to site and age as we became aware that ASA grading is highly subjective and has poor inter-rater reliability. We added the UCLA Activity Scale [28] as a secondary outcome measure to provide valuable information on the participant activity levels pre- and post-operatively. The HADS [29] was also added to be taken at baseline to inform the purposive sampling for the embedded qualitative study. Symptoms of anxiety and depression can impact the experience and perception of recovery. The embedded qualitative study was also simplified by the removal of the physiotherapists’ interview after agreeing that these would not add relevant information towards the outcome measure due to recall biases that would be introduced by practical aspects of running these interviews.

Further changes were made in June 2018 allowing further clarifications. This was done following the removal of the Body Mass Index (BMI) requirement enforced by one of our surgery sites. The associated exclusion criteria could, therefore, be removed opening the recruitment to a wider population and thus improving the representativeness of the study sample as many patients have a BMI greater than 35. In addition to this, the criteria excluding prior knee surgery was refined to exclude only previous surgery of the collateral ligaments of the knee as previous surgery on the cruciate ligaments would not affect the trial outcome as these ligaments are to be removed during surgery. The clarification of this exclusion criteria also permitted for previous non-intra-articular knee surgery (e.g. minor procedures around the knee) which were excluded despite not affecting the trial outcome. The visit windows were also reviewed as part of these changes to increase the baseline window from − 21 days to − 42 days up to surgery and to change the 6-month visit time-frame from ± 2 weeks to + 4 weeks. The former ensuring enough time for the assessments to take place before randomisation and the latter that all participants would have a full 6-month rehabilitation period before undertaking the last follow-up visits. Additional changes included the addition of the learning curve details for surgeon training to perform the intervention, the addition of the process for participants to be informed of their knee allocation at the end of the trial as part of the result dissemination, the clarification of the non-adherence and non-retention section to confirm that any data collected up to a participant withdrawal will be retained and the clarification of the safety reporting period and responsibilities. This amendment also allowed us to update the compliance section to add the General Data Protection Regulation (GDPR) [46].

Following on the previous amendment, additional modifications were made in August 2018 after the agreement that the recruitment of patients with previous TKR could be allowed as long as they are over a year old at the time of the consultation and painless, mildly or moderately painful. This was agreed to create a more representative dataset while ensuring that these participants’ mobility will not be affected by contralateral pain.

Additional changes were made in December 2018 to include the *maximum voluntary* isometric contraction (MVIC) of the hamstring and quadriceps muscles on both limbs to assess the known issue of muscle strength loss after TKR [47]. This biomechanical measure evaluates post-operative quadriceps and hamstring muscle-strength loss and subsequent recovery in both the non-operative legs and healthy control legs for comparison. The inclusion criteria were also amended to remove ‘Patient willing to provide full informed consent to the trial, including consent for any incidental findings to be communicated to their GP’. This does not need to be an inclusion criterion as a potential participant would not be enrolled on the trial if the consent form, which includes a statement about communicating findings with the GP, was not initialled and signed. In addition the PIS was amended to clarify that baseline data collected for participants that may not progress to randomisation or surgery, for reasons other than withdrawal, will be retained and used as observational data.

Furthermore, the protocol was amended in March 2019 to extend the 6–8-week visit window to 6–10 weeks to ensure that all participants can be seen within the appropriate window. An additional time point for collecting changes in pain medication was also added to the participant timeline at discharge from surgery. This will allow for a comparison between the participant-reported pain medications at the Week-1 telephone call and what was prescribed at discharge.

### Consent or assent

Potential participants will be provided with a PIS and given time to read it fully. Following a discussion with a medically qualified investigator or suitably trained and authorised delegate, any questions will be satisfactorily answered and if the participant is willing to participate, written informed consent will be obtained. During the consent process it will be made clear that the participant is free to refuse to participate in all or any aspect of the trial, at any time and for any reason, affecting their treatment.

Potential participants who, in the opinion of the clinical team do not have capacity to consent, will be ineligible for this study. If a participant loses capacity during the course of the trial, they will be withdrawn from the any further assessments but, the data which has already been collected will be retained.

Consent will be re-sought if new information becomes available that affects the participant’s consent in any way. This will be documented in a revision to the PIS and the participant will be asked to sign an updated consent form. These will be approved by the Ethics Committee prior to their use. A copy of the approved consent form is available from the NCTU trial team.

No additional consent will be sought for the collection or use of additional participant data or biological specimens as no such studies are planned.

### Confidentiality

Any paper copies of personal trial data will be kept at the participating site in a secure location with restricted access. Following consent, identifiable data will be kept on the trial database to allow the MoveExLab staff to contact participants to arrange appointments. Only authorised trial team members will have password access to this part of the database.

Confidentiality of a participant’s personal data is ensured by not collecting participant names on CRFs and limiting access to personal information held on the database at NCTU. At trial enrolment, the participant will be issued a PIN and this will be the primary identifier for the participant, with secondary identifiers of month and year of birth and initials.

The participant’s consent form will carry their name and signature. These will be kept at the trial site, and a copy sent to NCTU for monitoring purposes. They will not be kept with any additional participant data.

### Declaration of interests

The investigators named on the protocol have no financial or other competing interests that impact on their responsibilities towards the scientific value or potential publishing activities associated with the trial.

### Access to data

Requests for access to trial data will be considered, and approved in writing where appropriate, after formal application to the TMG. Considerations for approving access are documented in the TMG Terms of Reference. The Co-CIs and trial statistician at NCTU will have access to the full trial dataset.

### Dissemination policy

The results of the trial will be disseminated regardless of the direction of effect and will be reported following the Consolidated Standards of Reporting Trials (CONSORT) Statement [48]. Ownership of the data arising from the trial resides with the trial team. The publication policy will be in line with rules of the International Committee of Medical Journal Editors [49]. The TMG will decide on the dissemination strategy including presentations, publications and authorship.

## Discussion

This protocol describes a trial that will explore the performance and functional ability of two types of total knee implants by comparing them on multiple levels.

The use of validated PROMs as both primary and secondary outcomes will allow the comparison of the Journey II BCS and the Genesis II TKR implants in a standardised manner widely used in the literature. The addition of biomechanical, radiological, clinical efficacy and safety outcomes will permit an in-depth comparison of the implants and to fully assess the performance of both implants’ design in a comprehensive way. This will also highlight any relationships between each of these individual aspects and inform future study designs. The biomechanical outcome using everyday movement and detailed anatomical information from the rotational profile will both provide invaluable and pragmatic information on the knee implants in situ which will help clinicians in the investigation and management of participants before and after TKR. Additionally, the embedded qualitative study will investigate not only participant-related constructs associated with both their TKR and rehabilitation but also provide surgeon’s perspectives.

One of the challenges linked with the collection of varied outcome measures is the participant visit burden. This has been considered very carefully and the trial has been designed for study visits to be combined with routine clinical visits or to be undertaken over the telephone.

## Supplementary information

**Additional file 1.** Standard Protocol Items: Recommendations for Interventional Trials (SPIRIT) 2013 Checklist.

## Data Availability

Public access to the full trial protocol, trial-related documents, participant-level dataset and statistical code may be made on request to the TMG.

## Abbreviations

ADEs: Adverse drug events
AEs: Adverse events
BCS: Bi-Cruciate Stabilised knee arthroplasty
Co-CI: Co-chief investigator
Consort: Consolidated Standards of Reporting Trials
CoP: Centre of pressure
CRF: Case Report Form
CT: Computerised tomography
DMC: Data Monitoring Committee
EMG: Electromyography
FJS: Forgotten Joint Score
GCP: Good Clinical Practice
GDPR: General Data Protection Regulation
GISP3: General Information Security Policy 3
HADS: Hospital Anxiety and Depressions Score
HRA: Health Research Authority
ICH: International Council for Harmonisation
ISRCTN: International Standard Randomised Controlled Trials Number
MoveExLab: Movement analysis laboratory
mSEBT: Modified Star Excursion Balance Test
NCTU: Norwich Clinical Trials Unit
NERP: Norwich Enhanced Recovery Programme
NICE: National Institute for Health and Care Excellence
NNUH: Norfolk and Norwich University Hospital NHS Foundation Trust
OKS: Oxford Knee Score
OKS-APQ: Oxford Knee Score Activity and Participation Questionnaire
PI: Principle investigator
PIN: Participant Identification Number
PIS: Patient Information Sheet
PROMs: Patient-reported Outcome Measures
QA: Quality assurance
QC: Quality control
QMMP: Quality Management and Monitoring Plan
ROMs: Range of Movement
SAEs: Serious adverse events
SAP: Statistical Analysis Plan
TKR: Total knee replacement
TMG: Trial Management Group
TTB: Time-to-boundary
UKCRC: UK Clinical Research Collaboration

## References

[1] NICE. Osteoarthritis: care and management. Clinical Guideline [CG177]. Available at: https://www.nice.org.uk/guidance/cg177. Accessed 26 Jun 2019.

[2] National Joint Registry. StatsOnline Available at: http://www.njr.statsonline.org.uk Accessed 26 Jun 2019.

[3] Beswick AD, Wylde V, Gooberman-Hill R, Blom A, Dieppe P (2012). What proportion of patients report long-term pain after total hip or knee replacement for osteoarthritis? A systematic review of prospective studies in unselected patients. BMJ Open.

[4] Judge A, Arden NK, Kiran A, Price A, Javaid MK, Beard D, Murray D, Field RE (2012). Interpretation of patient-reported outcomes for hip and knee replacement surgery: identification of thresholds associated with satisfaction with surgery. J Bone Joint Surg.

[5] Zanasi S (2011). Innovations in total knee replacement: new trends in operative treatment and changes in peri-operative management. Eur Orthop Traumatol.

[6] Barrack RL, Schrader T, Bertot AJ, Wolfe MW, Myers L (2001). Component rotation and anterior knee pain after total knee arthroplasty. Clin Orthop.

[7] Hofmann S, Romero J, Roth-Schiffl E, Albrecht T (2003). Rotational malalignment of the components may cause chronic pain or early failure in total knee arthroplasty. Orthopade..

[8] Torga-Spak R, Parikh SN, Stuchin SA (2004). Anterior knee pain due to biplanar rotatory malalignment of the femoral component in total knee arthroplasty: case report. J Knee Surg.

[9] Berger RA, Rubash HE, Seel MJ, Thompson WH, Crossett LS (1993). Determining the rotational alignment of the femoral component in total knee arthroplasty using the epicondylar axis. Clin Orthop.

[10] Longstaff LM, Sloan K, Stamp N, Scaddan M, Beaver R (2009). Good alignment after total knee arthroplasty leads to faster rehabilitation and better function. J Arthroplast.

[11] Van Houten AH, Kosse NM, Wessels M, Wymenga AB (2018). Measurement techniques to determine tibial rotation after total knee arthroplasty are less accurate than we think. Knee..

[12] Cho Y, Lee MC (2014). Rotational alignment in total knee arthroplasty. Asia Pacific J Sport Med Arthrosc Rehabil Technol.

[13] Hirschmann MT, Konala P, Amsler F, Iranpour F, Friederich NF, Cobb JP (2011). The position and orientation of total knee replacement components: a comparison of conventional radiographs, transverse 2D-CT slices and 3D-CT reconstruction. J Bone Joint Surg.

[14] Brunner A, Eichinger M, Hengg C, Hoermann R, Brenner E, Kralinger F (2016). A simple method for measurement of femoral anteversion validation and assessment of reproducibility. J Orthop Trauma.

[15] Chan A-W, Tetzlaff JM, Altman DG, Laupacis A, Gotzsche PC, Krleza-Jeric K, Hrobjartsson A, Mann H, Dickersin K, Berlin J, Dore C, Parulekar W, Summerskill W, Groves T, Schulz K, Sox H, Rockhold FW, Rennie D, Moher D (2013). SPIRIT 2013 Statement: defining standard protocol items for clinical trials. Ann Intern Med.

[16] Dawson J, Fitzpatrick R, Murray D, Carr A (1998). Questionnaire on the perceptions of patients about total knee replacement. J Bone Joint Surg.

[17] Bohm ER, Loucks L, Tan QE, Turgeon TR (2012). Determining minimum clinically important difference and targeted clinical improvement values for the Oxford 12. American Academy of Orthopaedic Surgeons.

[18] Beard DJ, Harris K, Dawson J, Doll H, Murray DW, Carr AJ, Price AJ (2015). Meaningful changes for the Oxford Hip and Knee Scores after joint replacement surgery. J Clin Epidemiol.

[19] Williams DP, O'Brien S, Doran E, Price AJ, Beard DJ, Murray DW, Beverland DE (2013). Early postoperative predictors of satisfaction following total knee arthroplasty. Knee.

[20] Evans JT, Walker RW, Evans JP, Blom AW, Sayers A, Whitehouse MR (2019). How long does a knee replacement last? A systematic review and meta-analysis of case series and national registry reports with more than 15 years of follow-up. Lancet.

[21] Pennington M, Grieve R, Black N, van der Meulen JH (2016). Cost-effectiveness of five commonly used prosthesis brands for total knee replacement in the UK: a study using the NJR dataset. PLoS One.

[22] Moore C, Lenz N (2012). The evolution of guided motion total knee arthroplasty–The JOURNEY II Bi-Cruciate Stabilized knee system. Bone Joint Sci.

[23] Grieco TF, Sharma A, Dessinger GM, Cates HE, Komistek RD (2018). In vivo kinematic comparison of a bicruciate stabilized total knee arthroplasty and the normal knee using fluoroscopy. J Arthroplast.

[24] Smith TO, McCabe C, Lister S, Christie SP, Cross J (2012). Rehabilitation implications during the development of the Norwich Enhanced Recovery Programme (NERP) for patients following total knee and total hip arthroplasty. Orthop Traumatol Surg Res.

[25] Arshad H, Royan S, Smith TO, Barker L, Chirodian N, Wimhurst J (2014). Norwich Enhanced Recovery Programme vs. non-enhanced recovery following hip and knee replacement: a matched-cohort study. Int J Orthop Trauma Nurs.

[26] Dawson J, Beard DJ, McKibbin H, Harris K, Jenkinson C, Price AJ (2014). Development of a patient-reported outcome measure of activity and participation (the OKS-APQ) to supplement the Oxford Knee Score. Bone Joint J.

[27] EuroQol Group (1990). EuroQol—a new facility for the measurement of health-related quality of life. Health Policy.

[28] Zahiri CA, Schmalzried TP, Szuszczewicz ES, Amstutz HC (1998). Assessing activity in joint replacement patients. J Arthroplast.

[29] Zigmond AS, Snaith RP (1983). The Hospital Anxiety and Depression Scale. Acta Psychiatr Scand.

[30] Behrend H, Giesinger K, Giesinger JM, Kuster MS (2012). The ‘forgotten joint’ as the ultimate goal in joint arthroplasty: validation of a new patient-reported outcome measure. J Arthroplast.

[31] Nicholas MK, McGuire BE, Asghari A (2015). A 2-item short form of the Pain Self-efficacy Questionnaire: development and psychometric evaluation of PSEQ-2. J Pain.

[32] Podsiadlo D, Richardson S (1991). The Timed ‘Up & Go’: a test of basic functional mobility for frail elderly persons. J Am Geriatr Soc.

[33] Balke B (1963). A simple field test for the assessment of physical fitness. Civ Aeromedical Res Inst.

[34] Hertel J, Olmsted-Kramer LC, Challis JH (2006). Time-to-boundary measures of postural control during single leg quiet standing. J Appl Biomech.

[35] Kinzey SJ, Armstrong CW (1998). The reliability of the Star-Excursion Test in assessing dynamic balance. J Orthop Sport Phys Therap.

[36] Berger RA, Crossett LS, Jacobs JJ, Rubash HE (1998). Malrotation causing patellofemoral complications after total knee arthroplasty. Clin Orthop Relat Res.

[37] Jones CA, Voaklander DC, Suarez-Alma ME (2003). Determinants of function after total knee arthroplasty. Phys Ther.

[38] Johnson EC, Horwood J, Gooberman-Hill R (2014). Patients’ journeys through total joint replacement: patterns of medication use. Musculoskeletal Care.

[39] Johnson EC, Horwood J, Gooberman-Hill R (2016). Trajectories of need: understanding patients’ use of support during the journey through knee replacement. Disability Rehab.

[40] Merle-Vincent F, Couris CM, Schott AM, Conrozier T, Piperno M, Mathieu P, Vignon E (2011). Factors predicting patient satisfaction 2 years after total knee arthroplasty for osteoarthritis. Joint Bone Spine.

[41] Singh JA, O’Byrne M, Harmsen S, Lewallen D (2010). Predictors of moderate-severe functional limitation after primary total knee arthroplasty (TKA): 4701 TKAs at 2-years and 2935 TKAs at 5-years. Osteoarthitis Cartilage.

[42] Graneheim UH, Lundman B (2004). Qualitative content analysis in nursing research: concepts, procedures and measures to achieve trustworthiness. Nurse Educ Today.

[43] Lincoln YS, Guba EG (1985). Naturalistic inquiry.

[44] Silverman D (2000). Doing qualitative research: a practical handbook.

[45] Saklad M (1941). Grading of patients for surgical procedures. Anesthesiology.

[46] Information Commissioner’s Office. Guide to the General Data Protection Regulation. Available at: https://www.gov.uk/government/publications/guide-to-the-general-data-protection-regulation. Accessed 13 Dec 2019.

[47] Moon YW, Kim HJ, Ahn HS, Lee DH (2016). Serial changes of quadriceps and hamstring muscle strength following total knee arthroplasty: a meta-analysis. PLoS One.

[48] Schulz KF, Altman DG, Moher D, for the CONSORT Group (2010). CONSORT 2010. BMJ.

[49] ICMJE Recommendations Defining the Role of Authors and Contributors. Available at: http://www.icmje.org/recommendations/browse/roles-and-responsibilities/defining-the-role-of-authors-and-contributors.html. Accessed 26 Jun 2019.

